# Vitamins as Possible Cancer Biomarkers: Significance and Limitations

**DOI:** 10.3390/nu13113914

**Published:** 2021-11-01

**Authors:** Sascha Venturelli, Christian Leischner, Thomas Helling, Markus Burkard, Luigi Marongiu

**Affiliations:** 1 Institute of Nutritional Sciences, Department of Nutritional Biochemistry, University of Hohenheim, 70599 Stuttgart, Germany; sascha.venturelli@uni-hohenheim.de (S.V.); christian.leischner@uni-hohenheim.de (C.L.); thohelling@yahoo.com (T.H.); 2 Institute of Physiology, Department of Vegetative and Clinical Physiology, University of Tuebingen, 72074 Tuebingen, Germany

**Keywords:** vitamin A, vitamin B complex, vitamin C, vitamin D, vitamin E, vitamin K, cancer biomarker, cancer risk

## Abstract

The Western-style diet, which is common in developed countries and spreading into developing countries, is unbalanced in many respects. For instance, micronutrients (vitamins A, B complex, C, D, E, and K plus iron, zinc, selenium, and iodine) are generally depleted in Western food (causing what is known as ‘hidden hunger’), whereas some others (such as phosphorus) are added beyond the daily allowance. This imbalance in micronutrients can induce cellular damage that can increase the risk of cancer. Interestingly, there is a large body of evidence suggesting a strong correlation between vitamin intake as well as vitamin blood concentrations with the occurrence of certain types of cancer. The direction of association between the concentration of a given vitamin and cancer risk is tumor specific. The present review summarized the literature regarding vitamins and cancer risk to assess whether these could be used as diagnostic or prognostic markers, thus confirming their potential as biomarkers. Despite many studies that highlight the importance of monitoring vitamin blood or tissue concentrations in cancer patients and demonstrate the link between vitamin intake and cancer risk, there is still an urgent need for more data to assess the effectiveness of vitamins as biomarkers in the context of cancer. Therefore, this review aims to provide a solid basis to support further studies on this promising topic.

## 1. Introduction

The broadened food availability experienced by most countries after the Second World War, reflected by a higher energy intake and body weight in comparison to levels before the war, has fostered an ongoing epidemic of obesity that is a significant concern for public health worldwide [1,2]. Obesity has become so widespread that the American Medical Association categorized it as a disorder in 2013 [3]. One consequence of this pandemic of food overload is that malnutrition went into oblivion and nutrition-related issues are now often overlooked. It is well-known that the Western diet—which is characterized by high amounts of fat and protein but low in fibers (present in fruits and vegetables)—is essentially unhealthy because it leads to a higher cancer risk. For instance, Western countries show high prevalence of colorectal cancer [4]. Moreover, the 4-year survival rate in ovarian cancer has a hazard ratio (HR) of 2.28 (95% confidence interval: 1.34–3.89) in people on a Western diet, whereas it is 0.66 (0.43–1.01) in patients eating preferably vegetables [5]. Moreover, the Western diet increases the risk of developing cervical cancer precursors when compared to a diet rich in vegetables and fish (odds ratios, OR = 3.44) [6].

In addition to lacking fibers, the Western diet is meager in micronutrients, which includes vitamins and essential minerals [7]. This contradictory condition, characterized by a high food consumption but a depletion in micronutrient intake, has been defined as ‘hidden hunger’ and affects about two billion people around the globe [8]. For example, it has been estimated that about 75% of North Americans consume less than the recommended daily allowance (RDA) of folate [9], and 3–7% do not reach the RDA for vitamin C [10,11]. In Europe, it has been estimated that six out of eight countries show population-wide deficiencies of folate intake [12]. Another national survey in Germany indicated that 19% of men and 11% of women showed a folate intake below the RDA compared to 17% and 14% of Italian men and women, respectively [13]. Hidden hunger is preceded by the ‘nutrient gap’, defined as the deficiency of one or more micronutrients for a short period of time [14].

Vitamins are required not only for general cellular metabolism but also for good immunity [15]. As the immune system is engaged in eliminating infections, as well as fighting tumor formation [16,17], the hidden hunger not only worsens the quality of life of overweight and obese people (for instance, due to the morbidity caused by diabetes and heart diseases) but increases the risk of chronic inflammation and cancer. It should be considered that, for instance, a deficiency in vitamins B_6_/B_12_, can cause DNA damage just like the exposure to radiation or some chemical carcinogens [18]. Table 1 summarizes the RDAs and the recommended blood concentrations for the vitamins [19,20,21,22,23,24,25,26,27,28,29,30,31,32,33,34,35,36].

There are tantalizing results connecting diet to the risk of cancer. It is known that malnutrition (measured by body parameters such as weight and skeletal muscle index) fosters worse side effects in women undergoing chemoradiotherapy [37], but it is also known that people eating low amounts of fruits, vegetables, and vitamins are often more prone to persistent infections with human papillomavirus (HPV), the leading risk factor for the insurgence of cervical cancer [38,39]. Differences in blood concentrations of some micronutrients have been reported only in a subset of the studies. On the one hand, a case-control study of 111 patients with any cancer and 210 healthy controls reported concentrations of retinol, carotenoids, and vitamin E of 67.3 µg/dL, 114.5 µg/dL, 1160 µg/dL in the case group compared to 68.7 µg/dL, 111.6 µg/dL, and 1260 µg/dL in the control group [40], respectively. On the other hand, studies have confirmed the role of the Western diet in increasing the hazard of developing high-risk cervical lesions (odds ratio (OR) = 1.8), whereas a Mediterranean diet (defined by high intake of vegetables, legumes, fruits, nuts, cereals, fish, and unsaturated lipids) reduced the risk (OR = 0.4) [41].

The alteration of dietary habits or the direct administration of food supplements can significantly modify the blood and tissue concentrations of micronutrients. Intervention groups supplementing fruit extracts showed an increase in the blood α- and β-carotene without altering the expression of the oncosuppressor p27 and Ki-67, a marker of cell proliferation [42]. The supplementation of vitamins A, C, and E, as well as zinc and selenium, did not result in significant differences in blood biomarkers of inflammation in skin cancer patients (*n* = 34) in comparison to a placebo group (*n* = 26) [43]. The administration of vitamin B_3_ (niacin), vitamin C, selenium, zinc, and manganese to 23 volunteers daily over 4 months showed a decrease in TNF-α in comparison to the placebo group (*n* = 24, *t*-test *p*-value = 0.002), but not in IL-6, suggesting a moderate modulation of the inflammatory response [44]. A phase III clinical trial administering daily vitamin E (800 IU) and selenium (200 µg) for 3 years to a group of 156 volunteers reported a nonsignificant hazard ratio (HR) of 1.03 (0.67–1.60, log-rank *p*-value = 0.88) in preventing prostate cancer when compared to a placebo group (*n* = 147) [45]. The daily supplementation of vitamin A, C, and E together with selenium and zinc for 8 years in patients with a prostate-specific antigen below 3 µg/L reduced the risk of prostate cancer (HR = 0.88, 95% CI: 0.29–0.92, *p*-value < 0.001) when compared to the placebo group [46].

**Figure 1:**
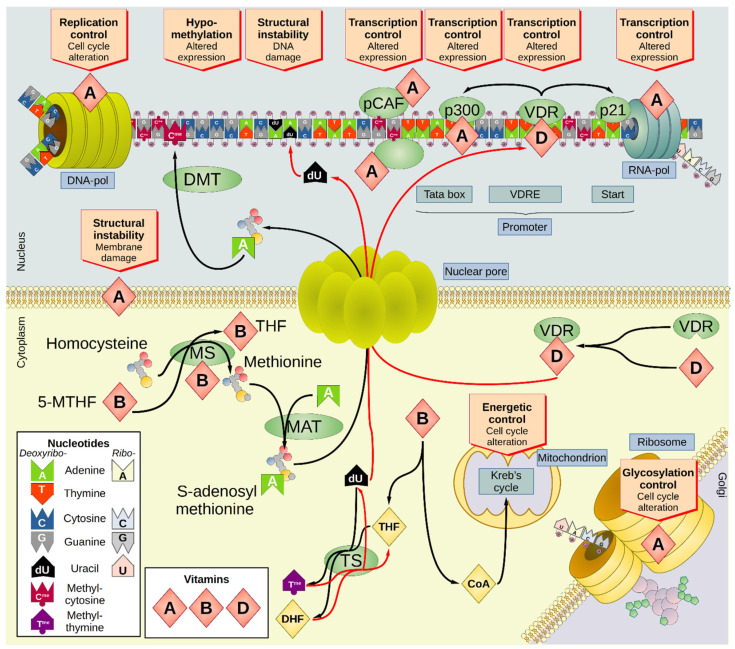
Overview of the impact of vitamins A, B complex, and D on the cellular biochemistry. The main effect of vitamin A, B complex, and D on cellular biochemistry is mostly due to the alteration of the transcriptional landscape of the cell, with relevant repercussions on the cell cycle. Vitamin A is involved in the replication and transcription processes but can also modulate the overall cellular expression by binding the transcription factor p300. Additionally, vitamin A modifies the genetic expression at the epigenetic level by modulating the histone acetyltransferase pCAF. Vitamin A is also involved in the glycosylation of proteins, in modulating important aspects of the cell cycle and cell-to-cell adhesion, and in the stability of the biological membranes. Vitamin B_5_ plays a relevant role in the metabolism of the cell, since it is incorporated in CoA that is involved in the Krebs cycle (TCA cycle) in the mitochondrial matrix. Additionally, vitamin B_9_ is converted to THF, which is then transformed into DHF by the enzyme TS simultaneously with the conversion of dU monophosphate into the methylated deoxythymidine monophosphate (T^me^). In depletion of vitamin B_9_, the reaction is shifted toward the reagents with the consequent accumulation of dU instead of T^me^ in the DNA. Since dU is more susceptible to physicochemical insults than the canonical nucleotides, it can generate DNA strand breaks, the accumulation of which increase the mutation rate of the cell, fostering oncogenesis. Vitamin B_9_ is also involved in the biosynthesis, mediated by 5-MTHF and MAT of methylated cytosine (C^me^), which is essential to the methylation of the DNA and the epigenetic modulation of the cellular expression. Deficiency in vitamin B_9_ is therefore associated with depletion of C^me^ and generalized hypomethylation. Vitamin D is bound by VDR that facilitates the translocation of the vitamin into the nucleus. The complex VDR/D binds to specific recognition sequences called VDREs within the promoters of several genes, including those involved in the regulation of the cell cycle (such as p21) and the epigenetic modulation (such as p300). CBP—CREB-binding protein; CoA—coenzyme A; CREB—cAMP response element-binding protein; DHF—dihydrofolate; DMT—DNA methyltransferase; DNA pol—DNA polymerase; dU—deoxyuridine; MAT—methionine adenosyl transferase; 5-MTHF—5-methyltetrahydrofolate; pCAF—p300/CBP-associated factor; RNA pol—RNA polymerase; TCA—tricarboxylic acid; THF—tetrahydrofolate; TS—thymidylate synthase; VDR—vitamin D recognition protein; VDRE—vitamin D response element.

**Figure 2:**
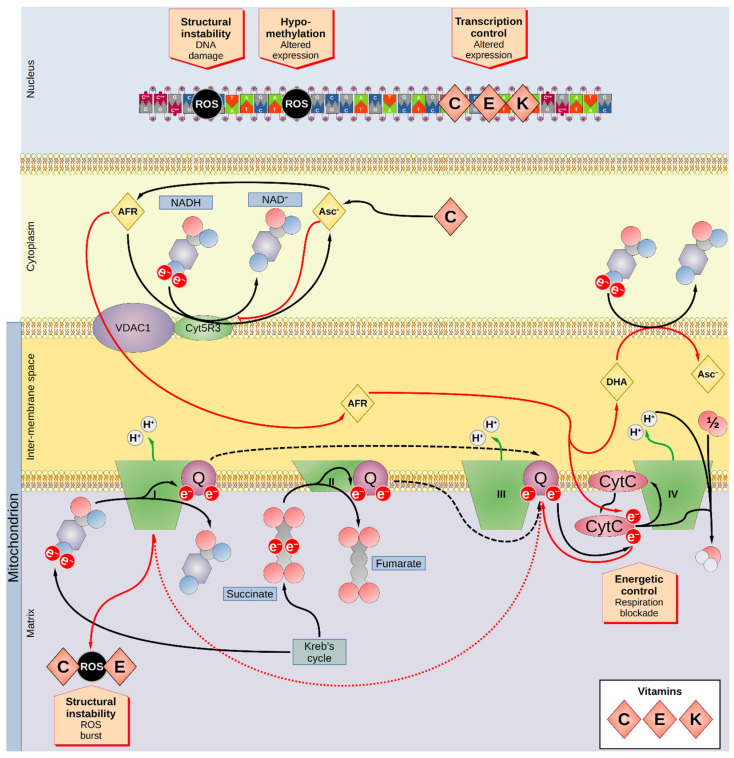
Overview of the impact of vitamins C, E, and K on the cellular biochemistry. Vitamins C and E as well as most minerals affect the cellular biochemistry mainly by altering the oxidative status of the cells. Vitamin C is converted rapidly to Asc^−^ and AFR. The Krebs cycle produces high energy molecules such as NADH, FADH_2_, ATP, or succinate. The Krebs cycle is linked to the respiration process that occurs mainly on the inner membranes of mitochondria, where several multiprotein complexes (I-IV) extract electrons from the high-energy molecules to pump protons (H^+^) into the intermembrane compartment of the mitochondria (third compartment of the figure). Protons are then pumped back into the cytoplasm producing ATP. In particular, complex I (NADH-ubiquinone oxidoreductase) oxidizes NADH to NAD^+^, and complex II (succinate-CoQ reductase) oxidizes succinate to fumarate, yielding FADH_2_. The acquired electrons are transferred to ubiquinone (also known as CoQ), which translocates them to complex III, which modulates the transfer of the electrons from CoQ to CytC, which then transfers them to complex IV (cytochrome c oxidase). Complex IV converts O_2_ to H_2_O in presence of H^+^. Under physiological conditions, the enzyme Cyb5R3 transfers one electron (e^−^) from NADH to AFR, producing Asc^−^ and NAD^+^. An excess of Asc^−^, however, inhibits Cyb5R3 and the Cyb5R3-associated membrane pump VDAC1 transfers AFR into the intermembrane compartment, which then transfers an electron directly to CytC, blocking the respiratory process. Incapable of reaching complex IV, the electrons in the transfer chain proceed back to complex I, producing a burst of ROS (dotted red arrow). Furthermore, AFR is converted into DHA that is then reduced to Asc^−^, producing NAD^+^. The newly produced Asc^−^ then further inhibits Cyb5R3 and the excess NAD^+^ can shift the balance of the reactions taking place in the Krebs cycle (red arrow). However, vitamins C and E act as scavengers neutralizing ROS. Vitamins C, E, and K can modulate the expression of several genes. ROS can directly damage the DNA chain, fostering oncogenesis, but can also influence the epigenetic control of genetic expression by inducing hypomethylation via 8-oxo-dG. AFR—ascorbyl free radical; Asc^−^—ascorbate; CoQ—coenzyme Q; Cyb5R3—NADH-cytochrome b5 oxidoreductase 3; CytC—cytochrome c; DHA—dehydroascorbate; e^−^—electron; FADH_2_—flavin adenine dinucleotide (reduced form); NADH—nicotinamide adenine dinucleotide (reduced form); 8-oxo-dG—8-oxo-deoxy-guanosine; ROS—reactive oxygen species; VDAC1—voltage-dependent anion-selective channel 1.

A better understanding of the link between nutrition and cancer risk might help to change the dietary behavior to prevent the insurgence of cancer. To comprehend the role of vitamins in oncogenesis, the current review will cover the most recent research quantifying the association between micronutrient concentrations in either blood or tissues and the risk of cancer in terms of risk ratios. The summary of the association between vitamins and cancer in the observational studies reviewed herein is reported in Table 2, while Figure 1 and Figure 2 visualize the role of vitamins on the cellular biochemistry. Since vitamins can be quantified with relative ease by employing noninvasive tests, the present work aimed to determine whether the selected molecules could potentially be applied as biomarkers for the early detection of cancer.

## 2. Vitamins

### 2.1. Vitamin A

Vitamin A (retinol) is present mainly in the liver, butter, whole milk, and cheese, but its precursor, β-carotene, is present in practically all fruits and vegetables [83,84]. Retinol is transported in the bloodstream bounded to the plasma retinol-binding proteins (RBPs) and then it is adsorbed by the retinol-binding receptors on the surface of the cells [85]. Intracellularly, retinol is converted into retinyl esters and, finally, to retinoic acid. Vitamin A is part of the visual pigment rhodopsin; thus, deficiencies of this micronutrient result in night vision impairment. Also, vitamin A is essential in embryogenesis and its intake must be carefully regulated since it has been shown to be teratogenic in high doses. Finally, retinol is indispensable for the cultivation of B lymphocytes [86], the growth of thymocytes [87], and the activation of natural killer lymphocytes [88], features that highlight the importance of vitamin A for the immune response.

The link between vitamin A and oncogenesis is complex. Animal models have demonstrated the anticancer activity of vitamin A [89,90], a feature backed up by epidemiological studies indicating how vitamin A deficiency was associated with a higher risk of cancer [91]. Thus, vitamin A has been the subject of extensive research in chemoprevention. Apart from the effects on immune cells, it has been shown that this micronutrient is involved in the structure of the cellular membrane, in the process of protein glycosylation, and in the regulation of the cell-to-cell adhesion [91]. Vitamin A also stimulates RNA transcription and DNA replication [92,93], and it has been suggested that retinoic acid binds to a complex containing the transcription factor p300 and the histone acetyltransferase p300/CBP-associated factor (pCAF) [85]. Unsurprisingly, then, the dysfunction of vitamin A is associated with the dysregulation of cellular differentiation [91].

A study of 966 prostate cancer cases and 1064 healthy controls did not show any significant differences in the blood concentrations of carotenes and retinol between these groups [47]. A case-control study with 142 prostate cancer patients and 142 controls reported an OR of 0.8 (0.4–1.5) related to blood concentrations of β-carotene, but without statistical significance (test for trends *p*-value = 0.33) [48]. However, the blood concentrations of vitamin A were reported to be in median 59.4 µg/dL in 84 prostate cancer cases compared to matched healthy controls (65.1 µg/dL), determining a relative risk (RR) of 2.4 for the ratio lower over the upper quartile of blood vitamin A in cancer cases [54]. A survey of 278 lung cancer cases and 483 matched healthy controls reported a significantly lower concentration of α-carotene in the former group (*t*-test *p*-value = 0.03) [55]. A longitudinal study reported that the blood concentration of β-carotene was 7.2 µg/dL in the cases of any cancer compared to 8.4 µg/dL in the controls (*t*-test *p*-value < 0.001) [56].

For retinol, a case-control study reported that the OR between the lower and upper quartiles was 0.4 for the development of prostate cancer, albeit with a slightly nonsignificant association (test for trends *p*-value = 0.07) [49]. Similarly, a study on 975 prostate cancer cases showed an OR of 1.30 (1.00–1.68) for the development of cancer and 1.74 (1.14–2.68) for the development of aggressive cancer when compared to people in the upper and lower quartiles of blood retinol concentrations [53]. The blood concentrations of retinol in 692 cases of prostate cancer and 844 matched controls did not show an increased risk of developing cancer (OR = 0.80, 95% CI: 0.57–1.11; test for trends *p*-value = 0.11) but higher concentrations of retinol were linked to a reduced risk of developing aggressive cancer (OR = 0.52, 95% CI: 0.32–0.84; test for trends *p*-value = 0.01) [50].

A comparison of prostate cancer cases (*n* = 1433) and controls (*n* = 1433) did not show any relation between blood concentrations of retinol and cancer [51]. A longitudinal study reported that the blood concentration of retinol at baseline was 64.5 µg/dL in 453 males who developed any type of cancer over eight years than the 66.7 µg/dL of 1419 matched healthy controls (*t*-test *p*-value < 0.01) [56]. Another longitudinal study reported the opposite trend, where higher retinol concentrations at baseline were observed in subjects who developed prostate cancer within three years than in healthy controls (HR = 1.19, 95% CI: 1.03–1.36, test for trends *p*-value = 0.009) [52].

### 2.2. Vitamin B Complex

The B vitamins represent a complex of water-soluble vitamins—thiamine (B_1_), riboflavin (B_2_), niacin (B_3_), pantothenic acid (B_5_), pyridoxine and pyridoxal (B_6_), biotin (B_7_), folic acid (B_9_), and cobalamins (B_12_)—that are present in a wide variety of animal and plant foods [94]. Specific receptors under the control of heterogeneous nuclear ribonucleoprotein E1 (hnRNP-E1) mediate cellular folate intake [95]. Tetrahydrofolate (THF) is the bioactive derivative of folic acid, which can transfer C1 units (methyl, methylene, formyl, formimino, and methenyl groups) with different oxidation states. First, dihydrofolate reductase (DHFR) catalyzes the two-fold reduction of folate via dihydrofolate (DHF) to THF. For example, thymidylate synthase (TS) is a highly conserved enzyme that transfers a methyl group from THF to deoxyuridine monophosphate (dUMP) to produce methylated deoxy-thymidine monophosphate (dTMP) and DHF [96]. Folate deficiency can affect the stability of the DNA by shifting the biochemical reactions carried out by TS toward an excess of dUMP, resulting in its incorporation into strands of DNA under replication or repair reactions [97]. Since uridine is more susceptible to chemical insult than thymidine, the affected DNA chains are prone to single and double-strand breaks, increasing the risk of mutagenesis and oncogenesis [98].

The increased DNA damage induced by folate depletion is applied in cancer therapy. Antifolate drugs are administered purposely to induce DNA insult in the fast-replicating cancer cells [99], highlighting the importance of this micronutrient for the chromosomal stability of the cells. On the other hand, the depletion of methylcytosine (C^me^) triggered by the depletion of vitamin B_9_ induces a global DNA demethylation that can foster oncogenesis [100]. It has been estimated that about 70% of human oncogenes are repressed by the presence of CG islands (CGI), a region of a high density of the duplex C^me^G [101]. Transposons are also highly methylated [102]. Therefore, a general demethylation status can promote the expression of both oncogenes and endogenous retroviruses, fostering genetic recombination and chromosomal instability. Instead, pantothenate (vitamin B_5_) is a constituent of coenzyme A, which is necessary for the synthesis and oxidation of fatty acids and the oxidation of pyruvate in the Krebs cycle [103].

Folate can also affect the cell environment indirectly by altering the infective process and, consequently, modulating the risk of cancer. For instance, cervical carcinoma is one of the most common causes of death and morbidity, ranking fourth among the causes of cancer-related deaths in industrialized countries and second in developing countries, respectively [104]. HPV is recovered in 99.7% of the carcinoma lesions, a feature that demonstrates the unique importance of this virus in the genesis of cervical carcinoma [105,106]. Folate deficiency reduces the translation of HPV’s minor capsid protein L2 through the action of hnRNP-E1 [107,108]. Consequently, the encapsidation phase of the HPV infection cycle cannot be completed, resulting in an accumulation of free viral genomes that increases the risk of viral integration and virus-driven oncogenesis [109].

A recent meta-analysis reported that high folate reduced the risk of lung cancer: the odds ratios of cases over healthy controls was 0.82 (0.74–0.90) in men, 0.70 (0.62–0.79) in former smokers, and 0.86 (0.75–1.00) in nonsmokers [57]. Low blood folate concentrations increased the cervical cancer risk (OR = 9.0) [58], while blood folate was lower in women with high-grade cervical lesions (14.3 nmol/L) than in women with low-grade lesions (15.9 nmol/L) and in healthy controls (18.2 nmol/L, 10.3–26.1); thus, folate concentrations below 14.1 nmol/L corresponded to an OR of 2.3 for developing low-grade lesions and 5.3 for the high-grade lesions [59]. Others have shown an OR of 2.7 for developing high-grade cervical lesions [60] and 1.68 for cervical cancer development in the presence of plasma folate below 3.19 ng/mL [61].

However, low folate concentrations were also associated with better prognostic value in B-cell lymphoma [64]. A comparison of 322 patients indicated that people in the lower tertile of blood folate concentration had lower overall survival than people in the upper tertile (HR = 0.181, 95% CI: 0.075–0.437; *t*-test *p*-value < 0.001). Vitamin B_2_, followed a similar trend: HR = 0.258 (0.117–0.569), *p*-value < 0.001. High folate concentrations, combined with methylation of the HPV-16 early promoter, were associated with reduced risk of developing cervical lesions. Folate concentrations above 14.3 ng/mL combined with viral methylation above 11% resulted in an OR of 0.3 for the development of high-grade cervical lesions [62].

High vitamin B_12_ concentrations were associated with a higher risk of myeloid leukemia (*n* = 308, OR = 19.2, 95% CI: 13.1–28.0; *t*-test *p*-value < 0.0001) and malignant lymphoid tumors (*n* = 1658, OR = 6.0, 95% CI: 4.7–7.6; *t*-test *p*-value < 0.0001) when compared to healthy controls (*n* = 136 and *n* = 970, respectively) [65]. Conversely, high vitamin B_12_ blood concentrations, combined with methylation of the HPV-16 early promoter, were associated with a reduced risk of developing cervical lesions. B_12_ concentrations above 406.6 pg/mL combined with viral methylation above 11% resulted in an OR of 0.4 for the development of high-grade cervical lesions [62].

Measurement of vitamin B_6_ in a cohort of 549 volunteers indicated that people with concentrations above 52.4 nmol/L were at lower risk of pancreatic cancer than people with concentrations below 20 nmol/L (OR = 0.46, 95% CI: 0.23–0.92; test for trends *p*-value = 0.048) [63].

### 2.3. Vitamin C

**Figure 3:**
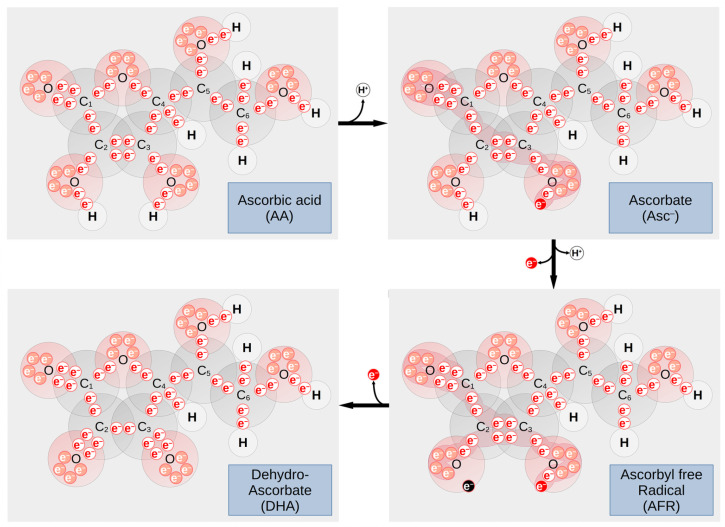
Overview of vitamin C chemistry. Due to its acidic properties, the majority of ascorbic acid is transformed into ascorbate by losing a proton in the C3-position under physiological conditions. Negative charge can be translocated among the molecule, stabilizing the ascorbate anion (the area of electron delocalization additionally marked in red color). The reducing agent ascorbate can be oxidized (e.g., by cellular NAD^+^ or metal ions in the plasma) resulting in the reactive intermediate AFR after losing one proton and one electron (the area of electron delocalization additionally marked in red color). AFR is further oxidized to the more stable DHA by losing another electron. The figure shows all the valence electrons of the oxygen atoms as small circles labeled with e^−^. Most important electrons for the presented reactions are highlighted in red color, while the unpaired electron of the free radical is marked black. Atomic radii of the elements are drawn in size ratio. For simplicity, data of the covalent radii for single bonds were used (H = 32 pm, C = 75 pm, O = 63 pm). AA—ascorbic acid; AFR—ascorbyl free radical; Asc^−^—ascorbate; C—carbon; DHA—dehydroascorbate; e^−^—electron, H—hydrogen; NAD^+^—nicotinamide adenine dinucleotide (oxidized form); O—oxygen.

Vitamin C is a water-soluble vitamin present in fruits and vegetables. Most animals can produce vitamin C in the liver and kidney, but due to a mutation in the L-gulonolactone oxidase humans rely on diet for its intake [110]. The protonated form of vitamin C is ascorbic acid (AA). Physiologically, vitamin C acts as a scavenger for radicals (e.g., hydroxyl OH^−^ or superoxide O_2_^−^), thus generating ascorbyl free radical (AFR) and hydrogen peroxide (H_2_O_2_) or water, respectively, or as a reducing cofactor (electron donor) in Cu(I)-dependent monooxygenases or Fe(III)-dependent dioxygenase reactions, as in prolyl- and lysyl-hydroxylases during collagen synthesis. Vitamin C donates its electrons sequentially, becoming first AFR and then dehydroascorbic acid (DHA) (Figure 3) [111]. As vitamin C is involved in the oxidative state of the cells, it can have an effect on the cells by damaging cellular structures (such as membranes and DNA) and impairing biochemical pathways. Both impairments aid in the insurgence of cancer.

Cancer tissues accumulate higher amounts of vitamin C than normal cells, a feature that is exploited as an anticancer treatment [112]. The mechanism by which vitamin C damages cancer cells is two-fold [110,113]: first, cancer cells have a reduced capacity to remove H_2_O_2_ and ROS; second, high concentrations of vitamin C boosts the production of ROS that, in turn, increases the redox activity of iron. Nevertheless, our group has demonstrated that the anticancer activity of vitamin C is dependent on the oxygen concentration, thus strengthening its link with the oxygen biochemistry [114]. We also demonstrated that the cytotoxicity of vitamin C requires high doses of this micronutrient (corresponding to the intravenous administration of grams of this vitamin) [115]. In addition, high physiological doses (200 µM), and especially pharmacological doses (8 mM), of vitamin C could profoundly alter the expression profile of interfering RNAs, an outcome whose impact on the affected cells is yet to be fully established [115,116].

The simultaneous administration of vitamin C and K_3_ induces cell death in a peculiar fashion that is distinguished from both necrosis and apoptosis [117]. Such an atypical cell death has been first described in the 1990s and has been named autoschizis [118,119]. The main features of autoschizis are the expulsion of the cytoplasm through organelles-free vesicles (unlike apoptosis) and the concentration of damaged organelles around the nucleus [117]. At the molecular level, there is no activation of caspases, as occurs during apoptosis, and the DNA fragmentation produces neither internucleosomal fragments (180–220 bp in length), as in apoptosis, nor a smear, as in necrosis, but rather random pieces cut by the DNAse II [120]. Unlike in apoptosis, autoschizis induces local inflammation and is believed to be an aberrant form of apoptosis [117].

It has been proposed that vitamin C, in the form of ascorbate, might have a protective value at low concentrations but enhance oxidative stress at high concentrations [121]. In the mitochondria, AFR accepts an electron from the reduced form of nicotinamide adenine dinucleotide (NADH) through the action of NADH-cytochrome b5 oxidoreductase 3 (Cyb5R3), becoming ascorbate. AFR is part of the normal aerobic respiration process at low doses but in cancerous cells there is the concomitant increased expression of vitamin C transporters and oxidative processes. The result is the accumulation of AFR with the consequent unbalance of the mitochondrial activity and production of reactive oxygen species (ROS). Apart from causing damage to the DNA and biological membranes, the ROS boosts the biosynthesis of 8-oxo-deoxy-guanosine (oxo-dG) [122], which recruits ten-eleven translocation (TET) proteins that induce demethylation by base excision repair [123,124]. It has been shown that the resulting demethylation inhibits cancer proliferation and boosts apoptosis [125]. Since vitamin C can be involved in the generation of the ROS via H_2_O_2_ production and its simultaneous reaction with Fe^2+^ during the Fenton reaction, while also counteracting the ROS, this vitamin can generate a dynamic equilibrium in the oxidative status of the cell.

Vitamin C up-regulates the tumor suppressors p53 and p21 so that the expression of these proteins balance demethylation [126]. Vitamin C is also linked to histone demethylation [115,127] and the targeting of the hypoxia inducible factor (HIF) proteins used by cancer to thrive in the oxygen-depleted environment of tumor masses for proteasomal degradation via proline and asparaginyl hydroxylases (HIF hydroxylases, HIFH), which belong to the family of iron-containing dioxygenases [128]. In the gastric tract, vitamin C prevents the development of N-nitroso compounds [129]. Vitamin C is also paramount in the biosynthesis of collagen, whose acute depletion leads to scurvy [130].

The quantification of vitamin C intake in African Americans (*n* = 17) and Native Americans (*n* = 18) indicated a significantly (*t*-test *p*-value < 0.005) higher value in the former group (198 mg daily) than in the latter (48 mg daily) [66]. Other markers were significantly higher in African Americans than in Native Americans (total fat, cholesterol, folate, iron, vitamin A, and zinc). Since African Americans have a 60 times higher risk of colorectal cancer than Native Americans, these micronutrient discrepancies strengthened the epidemiological link between diet and the risk of cancer. However, the overlap between different micronutrients impaired the assessment of specific vitamins or minerals to the oncogenesis.

The blood vitamin C was significantly lower (*t*-test *p*-value < 0.05) in prostate cancer patients (*n* = 32, mean concentratio*n* = 4 μg/mL) than in healthy controls (*n* = 40, mean concentratio*n* = 13 μg/mL) [67]. The quantification of blood vitamin C concentration in gastric cancer patients (*n* = 16, mean value 3.8 μg/mL) and healthy controls (*n* = 12, mean value 7.1 μg/mL) also showed a significant decrease (*t*-test *p*-value = 0.01) [68]. Vitamin C concentration also reflected such a decrease in gastric juice (3.2 µg/mL in gastric cancer patients compared to 18.2 µg/mL in healthy controls, *t*-test *p*-value = 0.001). Since the patients were concomitantly infected with *Helicobacter pylori*, it was proposed that the vitamin C depletion was due to this bacterium. *H. pylori* toxin can disrupt the transport of vitamin C in the gastric lumen [131], and the resolution of the infection is followed by the recovery of vitamin levels [132].

### 2.4. Vitamin D

Vitamin D is a group of sterol derivatives which have hormone-like functions. In humans, the most important members of this group are vitamin D_2_ (ergocalciferol), which is nonenzymatically formed in the skin by ultraviolet irradiation of 7-dehydrocholesterol, and vitamin D_3_ (cholecalciferol), which is also formed by ultraviolet irradiation of the plant sterol ergosterol [133]. The hormonally active forms result from dual hydroxylation in the liver and the kidney to form 1,25-dihydrocholecalciferol (calcitriol) and 1.25-dihydroxyergocalciferol (ergocalciferol), respectively. The final conversion to its active form can further occur in other loci such as the brain, the pancreas, in adipose tissue, the heart, the colon, and in immune cells (such as monocytes and macrophages) [134]. Vitamin D is required for proper bone formation via the pronounced generation of osteoclasts and increasing plasma Ca^2+^ concentrations but has also shown antitumoral and antimetastatic capabilities [135]. Conversely, a reduced vitamin D intake has been linked to a higher risk of developing cancer, particularly hepatocellular carcinomas [136]. Interestingly, it has been estimated that about nine-tenths of the tissue macrophages are present in the liver [137], suggesting that the macrophages might be heavily modulated by vitamin D. Moreover, vitamin D has been shown to reduce the expression of IL-6 in hepatocytes [138].

Vitamin D is carried in the bloodstream attached either to vitamin D binding proteins (VDBPs) or albumin. Then it is transported actively into the cells where it can reach the nucleus, acting as a transcription factor on promoters containing the vitamin D response element (VDRE) [139]. It has been estimated that the human genome contains 2776 VDREs spread across 229 genes [140], including important signal pathways components like signal transducer and activator of transcription (STAT) 1 and nuclear factor κ-B (NF-κB) kinase. As a result, vitamin D is involved in regulating the cell cycle [141], which explains its participation in oncogenesis. Experimental models in vitro and in vivo have suggested a possible anticancer activity for vitamin D, but the translation into clinical practice has given suboptimal results [142].

A study on 29 participants has suggested that the blood concentrations of vitamin D—specifically its derivative 1.25-hydroxyvitamin D (calcitriol)—were relatively stable: the within-subject coefficient of variation was 14.9% for 5 years (from a mean of 60.0 nmoL/L at the baseline to 56.6 nmoL/L at follow-up) and the Spearman rank correlation coefficient between the baseline and the end of the study was 0.53 [143]. Mono-hydroxylated vitamin D_3_ (25-OH-D_3_) was linked to the mortality risk of hepatocellular carcinoma (HCC): the comparison of patients with blood concentrations below and above 10 ng/mL resulted in an HR of 2.23, CI: 1.33–3.72 [78].

A case-cohort study of 547 colorectal, 634 breast, and 824 prostate cancer patients reported a significant decrease in colorectal cancer risk in people having high blood concentrations of vitamin D compared with those with the lowest concentrations (HR for the upper quintile over the lowest quintile was 0.71, 95% CI: 0.51–0.98) but not for breast cancer (HR = 0.98, 95% CI: 0.70–1.36) or prostate cancer (HR = 1.11, 95% CI: 0.82–1.48) [69]. A study of 95 healthy volunteers did not find any association between the blood concentrations of vitamin D and either prostate cancer antigen or total antioxidant concentrations [74]. Conversely, a comparison of 60 prostate cancer patients and 120 age-matched healthy controls showed a reduced risk of cancer in the presence of high concentrations of vitamin D (OR = 0.785, 95% CI: 0.718–0.858, *t*-test *p*-value < 0.05) [75]. A study of 1000 cases of prostate cancer and 1000 healthy controls reported an increased risk of cancer (OR = 1.56, 95% CI: 1.15–2.12, test for trends *p*-value = 0.01) in people with high blood vitamin D [144]. These results confirmed a previous survey of 234 cases and 234 healthy controls reporting that vitamin D not bound to DBP increased the risk of prostate cancer (OR = 5.01, 95% CI: 2.33–10.78, test for trends *p*-value < 0.0001) [145]. In pancreatic cancer, people with blood concentrations of vitamin D above 100 nmoL/L had an OR of 2.12 (1.23–3.64) compared to people with low concentrations [146]. Women in the upper tertile of vitamin D_3_ blood concentration (≥98 nmoL/L) had a higher risk of breast cancer than those with a concentration in the lowest tertile (≤76 nmoL/L), with an OR of 0.97 (0.75–1.25) [72]. Conversely, a study of 195 postmenopausal breast cancer patients indicated that women with low concentrations of blood vitamin D (<30 ng/mL) had a higher rate of high-grade tumors and metastases than women with higher concentrations [71]. The study also reported that low vitamin D was associated with the overexpression of the proliferation marker Ki-67. Similarly, a study of 50 breast cancer patients highlighted how women with vitamin D blood concentrations below 20 ng/mL had a higher risk of developing larger tumors (*t*-test *p*-value < 0.001) and worse overall survival (*p*-value = 0.026) than women with higher vitamin concentrations [70].

A survey of 5313 lung cancer cases and 5313 matched healthy controls did not show any increased risk of cancer (OR = 0.98, 95% CI: 0.91–1.06) [76]. Vitamin D was instead shown to be protective against thyroid cancer: a survey of 506 cases reported OR = 0.63 (0.40–1.00) with a test for trends *p*-value = 0.046 when comparing patients in the upper quartile (cut points for season-specific quartile: darker months December–May, above 39.0 nmoL/L; sunnier months June–November, above 58.6 nmoL/L) of blood vitamin D concentration with those of the lowest quartile (cut points for season-specific quartile: December–May, less or equal to 23.9 nmoL/L; June–November, less or equal to 36.1 nmoL/L) [77]. The significance was even higher when comparing the vitamin D binding protein concentrations (OR = 0.49, 95% CI: 0.32–0.77, *p*-value = 0.001).

The concentration of vitamin D was not directly associated with an increased risk of renal cancer, but people with higher concentrations of 25-OH-D_3_ not bound to the carrier protein DBP showed a slightly higher risk of cancer than people with lower concentrations of unbound vitamin 25-OH-D_3_ (OR = 1.61, 95% CI: 0.95–2.73, test for trends *p*-value = 0.09) [147]. The role of free vitamin D was also observed in bladder cancer [148]. These results contrast to previous analysis showing that high vitamin D blood concentrations were not associated with pancreatic cancer (OR = 1.45, 95% CI: 0.66–3.15) [149].

Vitamin D showed not only diagnostic but prognostic value. For example, in a cohort of 1666 breast cancer patients, women with vitamin D blood concentrations in the lowest tertile (≤16.8 ng/mL) had lower overall survival (HR = 0.54, 95% CI: 0.40–0.72, test for trends *p*-value < 0.001) than women in the upper tertile (≥25.1 ng/mL) [73]. The studies described make it clear that it depends on the type of cancer whether too low or too high blood levels of vitamin D are problematic.

### 2.5. Vitamin E

Vitamin E is a set of related isoforms (α-, β-, γ-, and δ-tocopherols, and α-, β-, γ-, and δ-tocotrienol) with antioxidant activities and present in seeds and vegetable oils [150]. Vitamin E acts as a scavenger protecting biological membranes from ROS insults, but it is also involved in immune regulation by inhibiting the NF-kB and STAT3 signal pathways [151], cell proliferation via the phosphoinositide 3-kinase (PI3K) pathway [152], and apoptosis [153]. In addition, like vitamin C, vitamin E reduces the accumulation of N-nitroso compounds in the intestine [129].

A case-control study with 142 prostate cancer patients and 142 controls reported an OR of 0.7 (0.3–1.5) related to blood γ-tocopherol but without statistical significance (test for trends *p*-value = 0.27) [48]. A comparison of prostate cancer cases (*n* = 1433) and controls (*n* = 1433) did not show any relation between blood vitamin E and cancer [51]. Conversely, two separate cohorts (one carried out in the period 1974–1996 and the other in the period 1989–1996) in the U.S. showed significantly lower blood γ-tocopherol in prostate cancer cases [79]. In the first cohort (CLUE I), the median γ-tocopherol blood concentration was 0.20 mg/dL in cases (*n* = 182) and 0.24 mg/dL in the controls (*n* = 364) (Wilcoxon signed-rank test *p*-value of 0.02), whereas in the second cohort (CLUE II) the values where 0.25 mg/dL in the cases (*n* = 142) and 0.29 mg/dL for the controls (*n* = 284) (*p*-value < 0.001). The blood vitamin E was shown to be significantly lower (*t*-test *p*-value < 0.05) in prostate cancer patients (*n* = 32, mean concentratio*n* = 5.2 µg/mL) than in healthy controls (*n* = 40, mean concentratio*n* = 14.2 µg/mL) [67]. A survey of 278 lung cancer cases and 205 prostate cancer cases, matched to 483 controls, reported a significantly lower concentration of α-tocopherol in lung (*p*-value = 0.02) and prostate (*p*-value = 0.03) cancer than in the control group [55].

### 2.6. Vitamin K

Vitamin K is a fat-soluble vitamin that is naturally available in dietary fat in two forms, K_1_ (phylloquinone, enriched in leafy vegetables) and K_2_ (menaquinone, present mostly in liver, milk, and fermented soy products), whereas a synthetic chemical analogue (K_3_, menadione) has been used as an antitumoral molecule [135,154]. The cytotoxic properties of vitamin K_3_ are due to the reactivity of the quinone moiety of this molecule, which generates ROS [155]. In combination with vitamin C, K_3_ induces autoschizis [117]. Even vitamin K_2_ shows antitumoral activity [156] but the process is understood to be linked to the alteration of the cell cycle at the transcriptional level and to disruption of the biochemistry of carboxylation [157]. In particular, vitamin K enhances the expression of protein kinase A (which in turn inhibits the factor Rho) and the inhibition of NF-κB by suppressing IκB kinase (IKK), thus affecting cell proliferation [158,159,160,161].

Vitamins K_1_/K_2_ take part in the carboxylation of glutamic acid to generate γ-carboxylglutamic acid, which is incorporated in the blood clotting factors II, VII, IX, X, protein Z, protein S, and protein C [162]. Deficiency in vitamin K fosters abnormal carboxylation of prothrombin generating des-gamma-carboxy prothrombin (DCP)—also known as prothrombin induced by vitamin K absence or antagonist-II (PIVKA-II))—which has been identified as a prognostic marker of HCC [163]. The increased expression of des-γ-carboxyl-glutamic acid in HCC is not directly due to a deficiency of vitamin K because these cells showed the same concentrations of this micronutrient as normal cells [164]. It has been proposed that the incapability of HCC cells in completing the carboxylation is not due to deficiency in vitamin K but rather to mutations in the receptors recognizing the complex vitamin K/lipoprotein that reduce the concentrations of this micronutrient in cancer cells, which can be restored by supplementation with vitamin K [165].

Serum DCP has been regarded as a useful HCC marker because it can be observed at a higher frequency in patients than α-fetoprotein (AFP), which is used historically as a diagnostic endpoint [80,166]. For instance, DCP above 0.1 µg/mL was observed in 48.2% of 112 HCC patients compared to 40.2% having AFP above 200 ng/mL [167], and 94.7% of 38 HCC patients had DCP above 0.1 µg/mL compared to 51.4% of 35 patients with AFP above 100 ng/mL [168]. Other surveys showed that 48% of 120 HCC patients had DCP above 0.1 µg/mL [169], 67% of 76 HCC patients had DCP above 300 ng/mL [170], and 74% of 70 HCC patients above 20 mU/mL [171]. DCP provided a risk ratio of 5.653 (95% CI: 2.015–15.861, *p*-value 0.001) for the insurgence of HCC compared to 3.159 (95% CI: 1.028–9.709, *p*-value 0.0447) provided by AFP [82]. The blood concentrations of DPC were measured at 64 arbitrary optical density units per liter (U/L) in 100 HCC patients and 3 U/L in 59 healthy controls [81].

## 3. Discussion

The role that nutrition plays in oncogenesis is only recently being unveiled. Hence, there is a rising interest in assessing the involvement of vitamins in DNA stability, epigenetic regulation, and immune response. Such an assessment is carried out by either measuring the blood vitamin concentrations in cancer cases and healthy controls (an avenue of research that might identify diagnostic or prognostic biomarkers) or by intervention studies where the supplementation of specific micronutrients might reduce the risk of cancer or its progression.

However, the studies reported in the present review were heterogeneous in terms of the quantitative assay and statistics applied, and the results showed a positive or negative association between vitamin concentration and the risk of cancer or showed no association at all. Several reasons might explain the contradictory data reported herein, including heterogeneity in the cohorts (for instance, due to ethnicity or behavior), fluctuation in blood measurements, and the disparateness in the experimental methods applied. It can be seen in Table 2 that several statistical approaches were used (for instance, OR or HR) and the comparison of groups differed between studies (for instance, quartiles versus tertiles). Moreover, there may be hidden trends that are difficult to identify using standard statistical methods. Given the overlap between cofactors, it is plausible to imagine that more complex models, probably aided by machine learning algorithms, will be required to identify the relationships between vitamins and cancer risk.

Another explanation for the discrepant results might reflect that vitamins are not directly implicated in the oncogenic process but indirectly as cofactors. For instance, the DNA damage caused by the processes reported in the present review can increase the mutation rate but the subsequent induction of the oncogenic pathway remains a stochastic event. Alternatively, the discrepancies in the results may be due to an unbalanced diet, such as the Western style, which impairs several and overlapping biochemical pathways, thereby making it difficult to pinpoint a single causative agent.

Despite the heterogeneity of the results reported herein, the present review highlighted some shared trends. First, the majority of cases indicated a negative relationship between vitamin concentration and the risk of cancer. That is, low concentrations of vitamins acted as cofactors for oncogenesis. This trend is consistent with the concept of hidden hunger: an unbalanced diet is depleted of micronutrients and, consequently, there is a disequilibrium in cellular biochemistry (namely in the oxidative status) that increases the risk of oncogenesis. Second, the relationships between vitamin concentrations and cancers depend on the affected organ. For instance, the vitamin B complex was low in virtually all cancer types except for lymphomas. Similarly, vitamin C was low in prostate and gastric cancer but high in colon cancer.

Since the cells in particular and the body in general have robust ways to keep homeostasis for vitamins and repair the damage to biomolecules, it is unlikely that the transient unbalances of these molecules will determine oncogenesis. On the contrary, it is the chronic unbalance of vitamins that can overwhelm the cellular environment. Moreover, the simple ingestion of multivitamin/mineral supplements does not guarantee to solve the hidden hunger, although they are useful in resolving temporary micronutrient gaps [14]. The corollary of this limitation is that the quantification of the vitamins at a single time point might have little diagnostic or prognostic value. Conversely, a longitudinal track of the assumed concentrations of vitamins might provide information regarding the likelihood of DNA insult due to oxidative stress or structural fragility and epigenetic aberration. It might be feasible for a person to quantify—using commercial machines akin to the cholesterol or glucose meters—the circulating blood concentrations of vitamins over time, thus generating a profile indicating whether particular vitamins or minerals are assumed at a suboptimal concentration. Such data could be related to the risk of DNA insult and, subsequently, help to pinpoint people at a higher cancer risk. Although blood concentration is routinely used to establish vitamin concentrations, it has been highlighted that the presence of vitamins in the blood does not exactly reflect the concentrations in the tissues [14]. Thus, using tissue concentrations rather than blood (serum or plasma) concentrations may provide more reliable quantification, albeit at the expense of more invasive sampling. Consequently, further advances in the quantification of vitamins in physiological conditions is urgently needed in combination with epidemiological and clinical studies to establish the link between hidden hunger and oncogenesis. Additionally, the implications of vitamin overdose are less well understood than deficiency, prompting the need for more research into the effects on human physiology, including on oncogenesis.

Another critical aspect of the relationship between nutrition and cancer is awareness. As reported above, cervical cancer is virtually all related to HPV infection, but nutrition is a cofactor. Recently, the World Health Organization has set the elimination of cervical cancer as a public health priority [172]. Such a goal will be achieved by combining vaccination, screening, and treatment [173]. Perhaps this elimination campaign should also include a comprehensive education program on nutrition. The added value of educating people to correct nutrition will be to reduce the incidence of cervical cancer and many other illnesses such as several types of cancer, diabetes, and heart diseases while improving the quality of life of millions of citizens. Hence, awareness of correct nutrition is one of the most effective approaches to overcome the pitfalls of the Western diet with its associated health problems.

## 5. Conclusions

Vitamins are involved in many essential cellular processes such as DNA replication or transcription. Therefore, vitamins also have a relevant influence on the development or prevention of malignant diseases. The use of vitamins in tumor diagnostics seems promising but is still largely unexplored and, in conclusion, the present review confirms the potential of vitamins as biomarkers. A large body of studies show that higher blood concentrations of vitamins were linked to both a lower and a higher risk of cancer. However, more data are needed to assess the effectiveness of these biomarkers and if they are suitable only for specific types of cancer. This also applies to the question of whether inappropriate vitamin blood and/or tissue concentrations are one of the major causes of certain types of cancer or to what extent they are also the consequence of an already existing tumor disease. In any case, the determination of circulating vitamin concentrations is a noninvasive and widely applicable analytical method with immense potential as a tool for improving diagnostic and prognostic statements.

## Figures and Tables

**Table 1:** Summary of the vitamins reported in the present review.

Vitamin	RDA [µg]	Blood Concentrations [µmol/L]	Source	References
A (retinol)	700–900	>1.05	Fruits, vegetables, liver, butter, milk	[24,28]
B_1_ (thiamine)	1100–1200	70–190 × 10^−3^	Meat, vegetables	[26,28]
B_2_ (riboflavin)	1100–1300	10.5 × 10^−3^	Milk, eggs, offal	[22,28,29]
B_3_ (niacin)	14,000–16,000	-	Liver, meat, peanuts, whole grain	[28,30]
B_5_ (pantothenic acid)	5000 *	1.6–2.7	Meat, eggs, nuts, avocados	[28,31]
B_6_ (pyridoxine)	1500	>3.0 × 10^−2^	Fish, liver, meat, cereals, nuts	[25,28]
B_7_ (biotin)	40 *	330 ng/L	Liver, mushrooms, eggs	[28,32]
B_9_ (folic acid)	250	≥0.01	Leafy vegetables, legumes, oranges	[20,28]
B_12_ (cobalamin)	2.4	>221 × 10^−6^	Meat, fish, milk, eggs, liver	[23,28]
C (ascorbic acid)	75,000–90,000	>50	Fruits, vegetables	[28,33]
D_2_ (ergocalciferol)	10–20	>50 × 10^−3^	Sun irradiation	[28,34]
D_3_ (cholecalciferol)				
E (tocopherol)	15,000	>12	Seeds, vegetable oils	[28,35]
K_1_ (phylloquinone)	70 **	1.45 × 10^−3^	Leafy vegetables, liver, milk, soy	[28,36]
K_2_ (menaquinone)				

* AI is used instead of RDA. ** 1 µg phylloquinone/kg body weight. AI—adequate intake; RDA—recommended daily allowance.

**Table 2:** Summary of the association between vitamins and risk of cancer.

Vitamin		Organ	Association *	Measure ^†^	Reference
A		Prostate	None	Qt	[47]
			None	OR	[48]
			None	OR	[49]
			None ^‡^	OR	[50]
			None	OR	[51]
			Positive	HR	[52]
			Positive	OR	[53]
			Negative	RR	[54]
		Lung	Negative	OR	[55]
		Any	Negative	Qt	[56]
B complex	B_9_ (folic acid)	Lung	Negative	OR	[57]
	B_9_ (folic acid)	Cervix	Negative	OR	[58]
	B_9_ (folic acid)		Negative	OR	[59]
	B_9_ (folic acid)		Negative	OR	[60]
	B_9_ (folic acid)		Negative	OR	[61]
	B_9_ (folic acid), B_12_ (cobalamin)		Negative	OR	[62]
	B_6_ (pyridoxine)	Pancreas	Negative	OR	[63]
	B_9_, (folic acid), B_2_ (riboflavin)	Blood	Positive	HR	[64]
	B_12_ (cobalamin)		Positive	OR	[65]
C		Colon	Positive ^§^	Qt	[66]
		Prostate	Negative	Qt	[67]
		Stomach	Negative	Qt	[68]
D		Colon	Negative	HR	[69]
		Breast	None	HR	[69]
			Negative	Qt	[70]
			Negative	Qt	[71]
			Positive	OR	[72]
			Negative	HR	[73]
		Prostate	None	Qt	[74]
			None	HR	[69]
			Negative	OR	[75]
		Lung	None	OR	[76]
		Thyroid	Negative	OR	[77]
		Liver	Positive	HR	[78]
E		Prostate	None	OR	[48]
			None	Qt	[51]
			Negative	Qt	[79]
			Negative	Qt	[55]
			Negative	Qt	[55]
		Lung	Negative	Qt	[55]
K		Liver	Positive	Qt	[80]
			Positive	Qt	[81]
			Positive	HR	[82]

* Positive: high vitamin concentration is linked to an increased risk of cancer; negative: low vitamin concentration is linked to an increased risk of cancer; none: no significance observed. ^†^ Cr—correlation; Qt—comparison of concentrations between groups; HR—hazard ratio; IR—incidence rate; OR—odds ratio; RR—relative risk. ^‡^ Inverse relation with risk of aggressive cancer. ^§^ Overlaps with other parameters (total fat, cholesterol, vitamins A and B_9_, iron, and zinc).

## Abbreviations

AA	ascorbic acid
AI	adequate intake
AFP	α-fetoprotein
AFR	ascorbyl free radical
Asc^−^	ascorbate
C	carbon
CBP	CREB-binding protein
CGI	CG islands
CoA	coenzyme A
CoQ	coenzyme Q
CREB	cAMP response element-binding protein
Cyb5R3	NADH-cytochrome b5 oxidoreductase 3
CytC	cytochrome c
DCP	des-gamma-carboxy prothrombin
DHA	dehydroascorbate acid
DHF	dihydrofolate
DHFR	dihydrofolate reductase
DMT	DNA methyl transferase
DNA pol	DNA polymerase
E^−^	electron
dTMP	deoxythymidine monophosphate
dU	deoxyuridine
dUMP	deoxyuridine monophosphate
FADH	flavin adenine dinucleotide (reduced form)
H	hydrogen
HCC	hepatocellular carcinoma
HIF	hypoxia inducible factor
HIFH	HIF hydroxylase
hnRNP-E1	heterogeneous nuclear ribonucleoprotein E1
HPV	human papillomavirus
HR	hazard ratio
IKK	IκB kinase
MAT	methionine adenosyl transferase
5-MTHF	5-methyltetrahydrofolate
NADH	nicotinamide adenine dinucleotide (reduced form)
O	oxygen
OR	odds ratio
8-oxo-dG	8-oxo-deoxy-guanosine
pCAF	p300/CBP-associated factor
PI3K	phosphoinositide 3-kinase
PIVKA-II	prothrombin induced by vitamin K absence or antagonist-II
RBP	retinol-binding protein
RDA	recommended daily allowance
RNA pol	RNA polymerase
ROS	reactive oxygen species
RR	relative risk
STAT	signal transducer and activator of transcription
TCA	tricarboxylic acid
TET	ten-eleven translocation
THF	tetrahydrofolate
TS	thymidylate synthase
VDAC1	voltage-dependent anion-selective channel 1
VDBP	vitamin D binding protein
VDR	vitamin D recognition protein
VDRE	vitamin D response element
